# Genome-wide analysis of histone modifications can contribute to the identification of candidate *cis*-regulatory regions in the threespine stickleback fish

**DOI:** 10.1186/s12864-024-10602-w

**Published:** 2024-07-11

**Authors:** Genta Okude, Yo Y. Yamasaki, Atsushi Toyoda, Seiichi Mori, Jun Kitano

**Affiliations:** 1 Ecological Genetics Laboratory, National Institute of Genetics, Yata 1111, Mishima, Shizuoka, 411-8540 Japan; 2 Comparative Genetics Laboratory, National Institute of Genetics, Mishima, Shizuoka, Japan; 3 Faculty of Economics, Gifu-Kyoritsu University, Ogaki, Gifu, Japan

**Keywords:** *Cis*-regulatory region, Epigenetics, Histone modification, Transposon, Stickleback

## Abstract

**Background:**

*Cis*-regulatory mutations often underlie phenotypic evolution. However, because identifying the locations of promoters and enhancers in non-coding regions is challenging, we have fewer examples of identified causative *cis*-regulatory mutations that underlie naturally occurring phenotypic variations than of causative amino acid-altering mutations. Because *cis*-regulatory elements have epigenetic marks of specific histone modifications, we can detect *cis*-regulatory elements by mapping and analyzing them. Here, we investigated histone modifications and chromatin accessibility with cleavage under targets and tagmentation (CUT&Tag) and assay for transposase-accessible chromatin-sequencing (ATAC-seq).

**Results:**

Using the threespine stickleback (*Gasterosteus aculeatus*) as a model, we confirmed that the genes for which nearby regions showed active marks, such as H3K4me1, H3K4me3, and high chromatin accessibility, were highly expressed. In contrast, the expression levels of genes for which nearby regions showed repressive marks, such as H3K27me3, were reduced, suggesting that our chromatin analysis protocols overall worked well. Genomic regions with peaks of histone modifications showed higher nucleotide diversity within and between populations. By comparing gene expression in the gills of the marine and stream ecotypes, we identified several insertions and deletions (indels) with transposable element fragments in the candidate *cis*-regulatory regions.

**Conclusions:**

Thus, mapping and analyzing histone modifications can help identify *cis*-regulatory elements and accelerate the identification of causative mutations in the non-coding regions underlying naturally occurring phenotypic variations.

**Supplementary Information:**

The online version contains supplementary material available at 10.1186/s12864-024-10602-w.

## Introduction

*Cis*-regulatory mutations often underlie adaptive phenotypic evolution [1–5], although the relative importance of mutations in protein-coding and non-coding regions may differ among traits and evolutionary time scales investigated [6]. Although there are many examples of amino acid-altering mutations underlying adaptive phenotypic variations [7], few cases occur in which causative *cis*-regulatory mutations for naturally occurring adaptive phenotypic variations have been identified, except in several recent studies [8–14]. In most cases, evidence for *cis*-regulatory mutations underlying adaptive divergence is obtained only by allele-specific gene expression analysis using heterozygous individuals [15, 16] or expression quantitative trait loci (eQTL) analysis using admixed populations/strains [17, 18]. One of the significant obstacles in identifying *cis*-regulatory mutations is the difficulty in detecting the locations of functional promoters and enhancers within non-coding regions.

In the nucleus, DNA binds to histones to form chromatin. Chromatin states can differ between the transcriptionally active and repressed sites [19–21]. Transcriptionally active enhancers and promoters show high chromatin accessibility, enabling transcription factors to bind to DNA. Furthermore, active and repressive *cis*-regulatory elements have specific histone modifications [22, 23]. Histones at transcriptionally active sites often undergo modifications in their active marks, such as monomethylation of histone H3 lysine 4 (H3K4me1) and trimethylation of histone H3 lysine 4 (H3K4me3), with the former being relatively common in active and primed enhancers and the latter in active promoters. In contrast, transcriptionally repressed sites such as facultative and constitutive heterochromatin are often marked by trimethylation of histone H3 lysine 27 (H3K27me3) and histone H3 lysine 9 (H3K9me3), respectively. Thus, profiling of histone modifications can help identify the locations of active and repressed *cis*-regulatory elements in non-coding regions.

Chromatin accessibility can be detected using the assay for transposase-accessible chromatin-sequencing (ATAC-seq), in which the Tn5 transposase can access DNA at open chromatin sites and add next-generation sequence (NGS) adaptors [24]. DNA with specific histone modifications can be enriched using antibodies against specific histone modifications followed by sequencing. Chromatin immunoprecipitation-sequencing (ChIP-seq) has been widely used for sequencing genomic regions enriched with specific histone modifications. A recently developed method called cleavage under targets and tagmentation (CUT&Tag) has several advantages, such as the requirement of a small number of cells and a higher signal-to-noise ratio, compared to that of other techniques including ChIP-seq [25]. In CUT&Tag, fresh cells are bound to Concanavalin A-beads and permeabilized, followed by antibody binding. The antibody is detected using a fusion protein composed of Protein A, which binds to the antibody, and Tn5 transposase, which adds NGS adapters to the DNA [25]. The resulting libraries can be subjected to NGS.

The threespine stickleback fish (*Gasterosteus aculeatus*) has adapted to diverse environments, providing an excellent model for investigating the genetic mechanisms underlying adaptation and ecological speciation [26–29]. Genome-wide gene expression analysis of divergent ecotypes of the stickleback has shown parallel evolution of gene expression, in which similar patterns of gene expression differences between ecotypes were observed in independent lineages [30–33], suggesting that some of the gene expression differences between ecotypes may be adaptive. Genome-wide allele-specific expression analyses and eQTL mapping have shown that *cis*-regulatory mutations play a substantial role in the divergence of gene expression between ecotypes [33–36]. *Cis*-regulatory mutations in particular genes have been shown to underlie adaptive morphological evolution [13, 14, 37–40]. However, even in these cases, causative mutations have been rarely identified except in a few cases [13, 14, 39]. Therefore, we applied CUT&Tag and ATAC-seq to the threespine stickleback and tested whether information on histone modifications can help identify possible *cis*-regulatory variants underlying gene expression differences between ecotypes.

## Materials and methods

### Preparation of NGS libraries

A laboratory stock of a Pacific Ocean marine ecotype of the threespine stickleback (*Gasterosteus aculeatus*), originating from the Bekanbeushi River in Akkeshi, Hokkaido [41], was used for the analysis. All fish were adults with developed gonads and were maintained under 10% seawater at 16 °C, with a photoperiod of L:D = 16:8 h. After euthanizing the fish with ethyl 3-aminobenzoate methanesulfonate (MS-222), tissues (brain, gills, liver, and/or gonad) were immediately dissected for NGS library preparation.

For the CUT&Tag experiment, three individuals were used for taking fresh tissues (Gacu1-Gacu3 in Table S1), while one fish (Gacu4) was used for taking frozen tissues. Because the tissue size from each individual is limited, we could not conduct all analyses on all individuals: Gacu3 and Gacu4 were used for H3K4me1; Gacu1-3 for H3K4me3; Gacu2-4 for H3K9me3; Gacu2-4 for H3K27me3; Gacu1 and Gacu2 for control IgG (see Table S2 for the details). To prepare CUT&Tag libraries, each freshly dissected tissue was immediately transferred to a 2 mL plastic tube with 1 mL L-15 medium (Sigma-Aldrich, St. Louis, MO, USA) containing 0.2% collagenase type V (FUJIFILM Wako Pure Chemical Corporation, Osaka, Japan). To test whether frozen samples could be used for CUT&Tag, freshly dissected tissues from one individual (Gacu4) were transferred to plastic 1.5 mL tubes and immediately frozen by soaking in 100% ethanol with dry ice. Approximately one minute later, each tube with frozen tissue was transferred to and stored at − 80 °C for approximately 6 months until use. After tissues were roughly minced with scissors, samples were incubated at 16 °C with vortexing for at least 2 h. The tissue was slowly pipetted up and down ad libitum using a 1 mL pipette tip with a widened bore. Each homogenized sample was filtered through a cell strainer with a mesh size of 100 μm. The obtained cell number was counted using a cell counter Scepter 2.0 with 60 μm sensors (Merck, Darmstadt, Germany). Totally, 500,000 cells or less were transferred to a 1.5 mL tube and subjected to CUT&Tag library construction using the CUT&Tag-IT Assay Kit (Active Motif, Carlsbad, CA, USA) following the manufacturer’s protocol.

In this study, the following five antibodies were used: a rabbit polyclonal antibody against H3K4me1 (Active Motif, Cat#39,298, Lot#02119002), rabbit monoclonal antibody against H3K4me3 (Cell Signaling, Beverly, MA, USA, Cat#9751 T, Lot#14), rabbit monoclonal antibody against H3K27me3 (Cell Signaling, Cat#9733 T, Lot#19), rabbit polyclonal antibody against H3K9me3 (Active Motif, Cat#39,162, Lot#30,220,003), and rabbit IgG (Cell Signaling, Cat#66362S, Lot#2) as a negative control (Table S1).

For ATAC-seq experiment, two fish were used (Gacu5 & Gacu6 in Table S1): Gacu5 was used for ATAC-seq of the brain and gill, while Gacu6 was used for ATAC-seq of the brain, gill, liver, and ovary. To prepare ATAC-seq libraries, each freshly dissected tissue was transferred to a 2 mL plastic screw cap tube containing zirconia beads and 1 mL ATAC Lysis Buffer of the ATAC-Seq Kit (Active Motif). The tube was then shaken at 4,000 rpm for 30 s using a Micro Smash (TOMY, Tokyo, Japan). Each homogenized sample was filtered through a cell strainer with a mesh size of 100 μm and transferred to a 1.5 mL plastic tube. After centrifugation at 500 × *g* at 4 °C for 5 min, the supernatant was removed, and the cell pellet was subjected to tagmentation reaction using the ATAC-Seq Kit by following the manufacturer’s protocol. The prepared CUT&Tag and ATAC-seq libraries were sequenced using Illumina HiSeq2500 (Illumina, San Diego, CA, USA) in 100 bp × 2 and 50 bp × 2 paired-end modes, respectively. All sequenced reads were deposited in DDBJ (PRJDB17468). Sample information and accession numbers are listed in Table S2.

### Peak detection of CUT&Tag and ATAC-seq

We trimmed the adapter and low-quality sequences from the raw fastq files using Trimmomatic v. 0.39 [42] with the following parameters: LEADING:3, TRAILING:3, SLIDINGWINDOW:4:15, MINLEN:36. The trimmed reads were mapped to the reference genome of the threespine stickleback (v.5 assembly) [43] using Bowtie2 v. 2.5.1 [44] with default parameters. Peak calls were performed using macs2 v. 2.2.7.1 [45] with the following options: –BROAD, –broad-cutoff 0.1, -f BAMPE. Called peaks were annotated using annotatePeaks.pl in Homer v. 4.11 [46] with default parameters. In this study, we classified peaks into the following five categories based on the location of the peak center: (1) exon, (2) intron, (3) promoter or transcription start site (TSS) (− 1 kb to + 100 bp of TSS), (4) transcription termination site (TTS) (− 100 bp to + 1 kb of TTS), and (5) intergenic region (counted only for the closest gene), representing all peaks that did not fall into the above four categories. The number of peaks detected in each category is listed in Table S2.

For visualization of mapped short reads, BAM files were converted to bigWig format using deepTools v.3.5.4 [47] with the option of –minMappingQuality 10. Subsequently, mapped short reads and gene annotations were visualized using the Integrative Genomics Viewer (IGV) v.2.16.2 [48]. For cluster analysis of CUT&Tag and ATAC-seq data, read coverage for each 5,000 bp genomic window was calculated across the genome using multiBamSummary of deepTools v.3.5.4 [47] with -e –binSize 5000 options. Spearman’s correlation coefficients of read coverages were calculated and the heatmap was drawn using the plotCorrelation of deepTools v.3.5.4 [47].

### Analysis of the association between histone modifications and gene expression levels or evolutionary rates

To investigate whether histone modifications are associated with gene expression levels, we used RNA-sequencing (RNA-seq) data from various adult tissues of the same threespine stickleback strain derived from our previous study [49]. Sample information and accession numbers used in this study are listed in Table S3. Transcriptome analysis was conducted as described previously [50]. Adapter and low-quality sequences were removed using Trimmomatic v. 0.39 [42] with the following parameters: LEADING:3, TRAILING:3, SLIDINGWINDOW:4:15, MINLEN:36. The trimmed reads were mapped to the transcripts of the threespine stickleback reference genome (v.5 assembly) [43] using Salmon v. 1.10.1 [51] with default parameters, whereby transcript expression levels were estimated as transcripts per million (TPM) values. The sum of the TPM of all the isoforms was calculated for each gene and used for subsequent analyses. All genes were classified into the following three categories: genes with peaks within the promoter, exon, intron, and/or TTS regions (“Gene” in the figure), genes with peaks only in the intergenic regions (“Intergenic” in the figure), and genes without any surrounding histone modification peaks (“No peaks” in the figure). The expression levels were compared among these categories using the Wilcoxon rank sum test.

To investigate whether histone modifications were associated with protein sequence evolution, we used data on the maximum likelihood estimation of the pairwise non-synonymous/synonymous mutation rates (dN/dS) between *G. aculeatus* and a closely related species *Gasterosteus nipponicus.* The dN/dS data were obtained from our previous study [49, 52]. Because we may overestimate dN/dS when dS is close to 0, we excluded genes with dN/dS ≧ 99, as described previously [49, 52]. Because the dN/dS ratio was calculated using a previous version of the reference genome in Ensembl (BROAD S1), several genes annotated only in the v.5 assembly were not included in this analysis. All statistical analyses were conducted using the R v. 4.3.2 [53].

### Association between histone modifications and nucleotide variations

Next, we investigated whether different histone modifications were associated with nucleotide variation within populations (*π*) and nucleotide diversity between populations (*D*_XY_). Short reads of whole genome sequences of eight individuals from a stream population of the threespine stickleback from Asutani, Gifu, Japan, and 10 individuals from the Pacific Ocean marine population from the Bekanbeushi River, Hokkaido, Japan, were obtained from our previous studies [52, 54]. The accession numbers and sample information are listed in Supplementary Table S4.

For the quality filtering of raw fastq files, fastp v. 0.23.0 was used [55]. We first removed PCR duplicate by -D option, then applied following options to remove low quality reads and bases: –detect_adapter_for_pe –cut_right –cut_window_size 4 –cut_mean_quality 20 -l 35. The trimmed reads were mapped to the reference genome of the threespine stickleback (v.5 assembly [43]) using bwa-mem2 v. 2.2.1 [56] with default parameters. After mapping, multi-mapped reads were removed by using XA:Z and SA:Z flags in bam files. SNPs were called using BCFtools v. 1.9 [57] with the consensus caller mode. Filtering was then performed using vcftools v. 0.1.16 [58] with the following options; –max-meanDP 54.40642 –max-missing 0.8 –minQ 30 –minDP 8 as described previously [59]. Consequently, 8,278,733 SNPs were subjected to the subsequent analyses. We calculated *π* and *D*_XY_ in non-overlapping 10 kb windows using the all-site dataset with popgenWindow.py script (https://github.com/simonhmartin/genomics_general) [60]. Windows with fewer than 10 SNPs were excluded from analyses.

### Identification of possible *cis*-regulatory variants responsible for gene expression difference between the stream and marine ecotypes

Next, we examined whether histone modification data are useful for identifying possible *cis*-regulatory variants responsible for gene expression differences between the stream and marine ecotypes. First, to screen for genes that exhibited substantial differences in gene expression between ecotypes, we conducted RNA-seq using the lab-raised Pacific Ocean marine ecotype, which is the same strain used for chromatin analysis, and a lab-raised stream ecotype derived from a nearby site of the Asutani stream, a small stream and a pond in Gifu-kyoritsu University, Gifu, Japan. All the fish were reared at 16 °C under a 16:8 h photoperiod. These fish were first maintained at a low concentration of salinity (freshwater condition), as the fish needs a small amount of salt, we used 1% seawater (approximately 0.35 ppt) made with Instant Ocean (Aquarium Systems, Sarrebourg, France) and active carbon-filtered tap water. At the age of approximately two months old, half of the marine fish were gradually transferred to 100% seawater (seawater condition): 10% seawater for 1 d, 30% seawater for 3 d, 50% seawater for 2 mo, and 100% seawater (approximately 35 ppt) for 2 mo. Stream fish were reared only under freshwater conditions because they could not survive well in 100% seawater [61]. After euthanizing the fish with ethyl 3-aminobenzoate methanesulfonate (MS-222) at approximately six months of age, the gills were immediately dissected and stored in RNAlater (Thermo Fisher Scientific, Waltham, MA, USA).

Total RNA was extracted using the RNeasy Mini Kit (Qiagen, Hilden, Germany). For RNA-seq, we used four Pacific Ocean marine fish under freshwater conditions, four Pacific Ocean marine fish under seawater conditions, and four stream fish under freshwater conditions. Sequencing libraries were constructed using the NEBNext Ultra RNA Library Prep Kit (New England Biolabs, Ipswich, MA, USA). The prepared RNA-seq libraries were sequenced using an Illumina HiSeq2500 in the 100 bp × 2 paired-end mode. The accession numbers and sample information are listed in Supplementary Table S3.

Trimming and mapping of short reads were performed as described above. To investigate the overall gene expression differences between ecotypes, we first conducted a principal component analysis (PCA) of the TPM values of all genes using R v. 4.3.2 [53]. Next, we screened for genes specifically expressed in the marine ecotypes. To this end, we applied three criteria: (1) the average TPM value of four 1% seawater-acclimated marine fish exceeded eight times that of four 1% seawater-acclimated stream fish to pick up considerably higher expressed genes in marine fish than in stream fish, (2) the minimum TPM value of four 100% seawater-acclimated marine fish was above 10 to exclude the genes with low expression levels in marine fish, in which CUT&Tag experiments were conducted, and (3) genes were annotated on the assembled chromosomes of the v.5 reference genome of the threespine stickleback. Because we did not conduct chromatin analyses in the stream ecotype, we focused on the genes that are specifically expressed in the marine ecotype.

As we identified three possible ecotype-specific *cis*-regulatory insertions and deletions (indels) (see Results), these indels were confirmed via Sanger sequencing of PCR products, using the following PCR primers: *calcium uniporter regulatory subunit MCUb*, forward: TGA CGC GTA AAC AAA TCA GC, reverse: CTC TGG GAG AGG AAC AGA CG; *methyltransferase-like protein 27*, forward: TGG AAC GGT TTT CAT TCA TTT, reverse: AAA AGC AGA TTT CAC CGA TTG; lncRNA, forward: TGC TGC TAA ACG ATC ACT CG, reverse: TTC ACG GAC AAT CAA ACC AA. These primers were designed using Primer 3 v. 0.4.0 [62]. KOD One PCR Master Mix (TOYOBO, Osaka, Japan) was used for PCR with the following reactions: 35 cycles of 98 °C for 10 s, 55 °C for 5 s, and 68 °C for 5 s (lncRNA) or 25 s (other two genes). The PCR products were subjected to gel electrophoresis on a 1% agarose gel in TAE buffer. Four wild-caught individuals per ecotype were analyzed: the same Japanese Pacific Ocean marine population and the Gifu-kyoritsu stream population. A stream ecotype-specific insertion around *methyltransferase-like protein 27* gene was determined using Sanger sequencing after cloning into the pGEM-T Easy Vector (Invitrogen, Carlsbad, MA, USA). The deleted and inserted sequences were analyzed for sequence similarity to transposable elements using Repbase [63].

## Results

### Peak detection of histone modifications

**Fig. 1:**
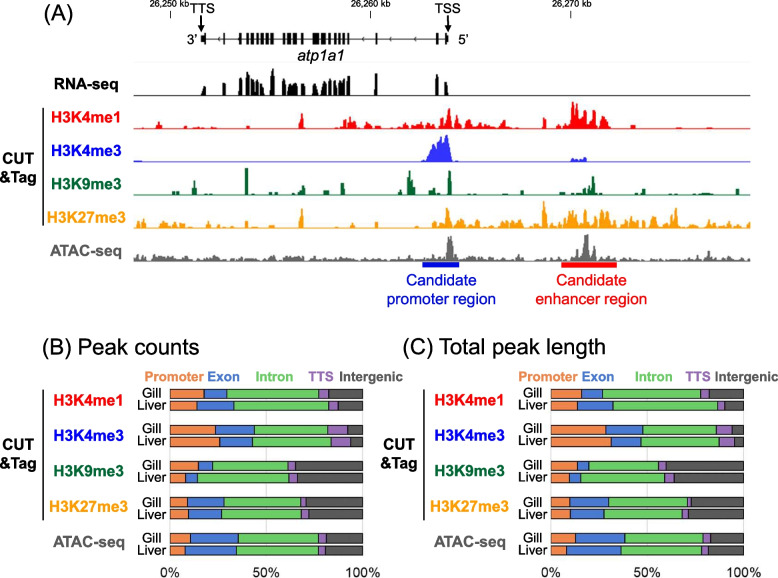
Peaks of CUT&Tag and ATAC-seq in a Japanese Pacific Ocean marine population of the threespine stickleback. **A** Visualization of peaks of RNA-seq, CUT&Tag, and ATAC-seq around the major isoform of the *atp1a1* gene on Chromosome 1. In the upper gene model, black boxes indicate exons, whereas black lines indicate introns. TSS indicates the transcription start site. At the bottom of the panel, a blue line indicates a candidate promoter region with H3K4me3 marks, whereas a red line indicates the candidate enhancer region with H3K4me1 marks. Data on the RNAseq, CUT&Tag, and ATAC-seq derived from the data on the gill of Marine-SW1, Gacu3, and Gacu6, respectively, are shown. **B** Proportions of peak counts in different genomic regions: promoter, exon, intron, TTS, and intergenic regions. While data on the gill and liver of Gacu3 data are shown for CUT&Tag, those of Gacu6 are shown for ATAC-seq. Other data is shown in Fig. S1. **C** Proportions of peak length in different genomic regions: promoter, exon, intron, TTS, and intergenic regions. The same data are used as in Fig. 1B. Other data are shown in Fig. S1

To examine whether CUT&Tag was successful, we first visually inspected the sequenced short reads mapped to the major isoform of the *atp1a1* gene, which encodes Na^+^/K^+^-ATPase (ENSGACG00000014324; NCBI Gene ID: 120,823,906; XM_040184383.1). This gene was selected as a representative example, because it is expressed in the gills and plays a crucial role in fish osmoregulation [64–66]. Clear H3K4me3 peaks were identified around the TSS (Fig. 1A), consistent with previous findings that H3K4me3 marks are often associated with active promoters [22, 23]. The H3K4me1 peaks were localized near the TSS and approximately 6–8 kb upstream of the TSS (Fig. 1A). Given the previous findings that H3K4me1 is often enriched in active and primed enhancers [22, 23], the region indicated by the red line in Fig. 1A is likely an enhancer. ATAC-seq indicated that open chromatin regions overlapped with the TSS and H3K4me1 peaks, supporting the idea that these are a promoter and an enhancer, respectively. H3K27me3 marks were broadly distributed upstream of the TSS. As H3K27me3 repressive marks co-localized with H3K4me1 marks, this gene may exhibit a bivalent state, which is important for rapid switching of gene expression [67], or may not be expressed in all types of cells in the gill.

Subsequently, to confirm that the peak calls of CUT&Tag worked throughout the genome, we analyzed the genome-wide patterns of histone modifications (Fig. 1B, Fig. 1C, Fig. S1). The peaks of different histone modifications were enriched in different regions of the genome. The H3K4me3 peaks were more concentrated in the promoter region than the peaks of other histone modification types. The H3K9me3 peaks were less abundant in the exons and more abundant in the intergenic regions compared to the peaks of other histone modification types. H3K4me1 and H3K27me3 displayed relatively similar patterns, although the H3K27me3 peaks were more enriched in intergenic regions than the H3K4me1 peaks. Overall, these patterns were consistent across various tissues and individuals (Fig. S1).

**Fig. 2:**
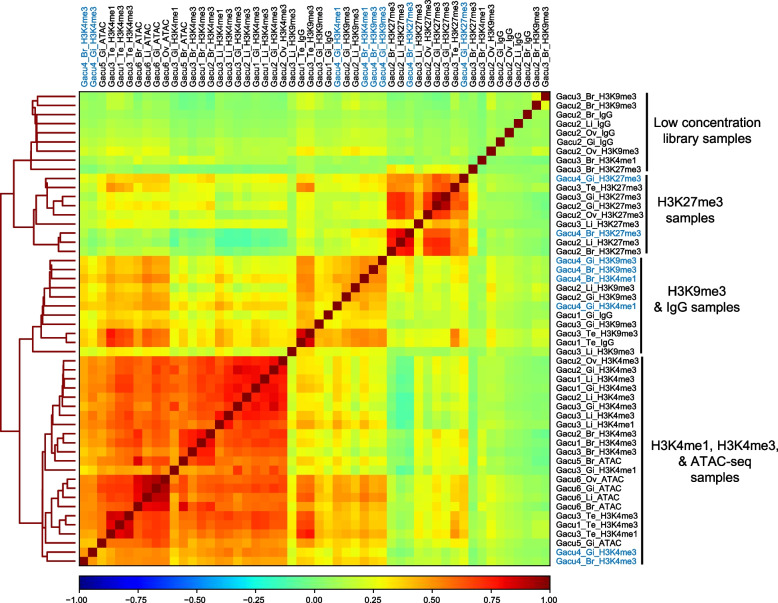
Clustering analysis of CUT&Tag and ATAC-seq data. Heatmap and dendrogram using Spearman’s correlation coefficients are shown. Colored squares indicate the Spearman’s correlation coefficients (see the scales below). Sample names with blue letters are frozen samples

Additionally, we compared genome-wide distribution of sequenced reads among samples (Fig. 2). Overall, samples with the same antibodies tended to cluster together. However, several samples whose library concentrations were under 0.26 nM clustered together (see the upper 9 samples of Fig. 2), indicating that libraries with low concentrations did not give rise to informative peaks. H3K9me3 samples clustered together with IgG negative control samples, indicating that CUT&Tag with H3K9me3 did not work well. Finally, for the frozen samples, although Fig. 1 suggests that the reads from the frozen samples looked similar to those from fresh samples, additional cluster analysis using only H3K4me3 samples showed that frozen samples make a distinct cluster from fresh ones (Fig. S2). These results indicate that caution is needed for using the data from the frozen samples and further experimental validation and optimization is necessary for the use of frozen samples.

### Association of histone modifications with gene expression levels

**Fig. 3:**
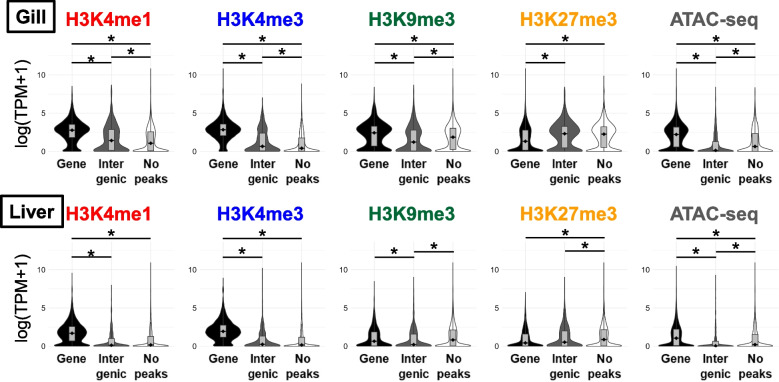
Association between histone modifications and gene expression levels. All genes were classified into the following three categories: genes with peaks within the promoter, exon, intron, and/or TTS regions (“Gene” in the figure), genes with peaks only in the intergenic regions (“Intergenic” in the figure), and genes without any surrounding histone modification peaks (“No peaks” in the figure). Asterisks indicate *P* < 0.001 in the Wilcoxon rank sum test. Gill and liver data from Gacu3 are shown, whereas other data are shown in Fig. S3. The Y-axis indicates the natural logarithm of TPM plus 1

To confirm that histone modifications are associated with the expression levels of nearby genes, we tested whether expression levels differed among (1) genes with peaks at the gene (either in the promoters, exons, introns, or TTS); (2) those with peaks only in nearby intergenic regions; and (3) those without any nearby peaks. Genes with H3K4me1 or H3K4me3 peaks at the gene exhibited significantly higher expression than those without these histone modifications (Fig. 3, Fig. S3). Similarly, the ATAC-seq data showed that genes in the open chromatin regions were expressed at higher levels than those in the closed chromatin regions (Fig. 3, Fig. S3). In contrast, genes with H3K27me3 peaks showed lower expression than those without H3K27me3 peaks. The relationship between H3K9me3 and gene expression levels varied across samples, consistent with the above data indicating that CUT&Tag analysis using H3K9me3 antibody in this study did not work well. When genes with peaks in promoters, exons, introns, and TTS were analyzed separately, we found similar trends, indicating that genes with H3K4me1, H3K4me3, and ATAC-seq peaks were expressed at higher levels and genes with H3K27me3 peaks were expressed at lower levels compared to those without peaks (Fig. S3).

### Association of histone modifications with sequence evolution

**Fig. 4:**
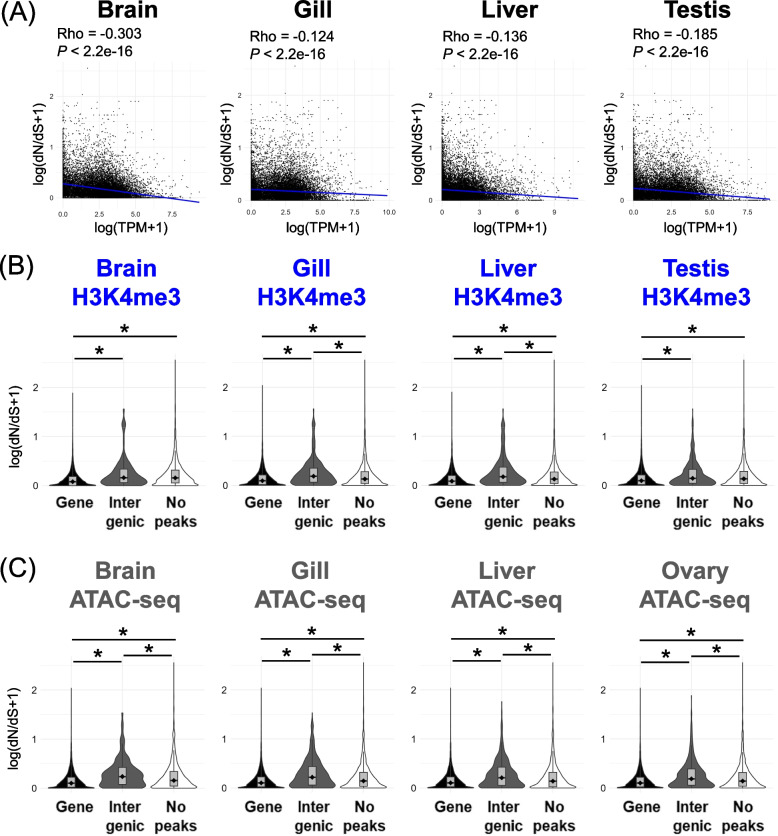
Association between histone modifications and dN/dS. **A** Negative correlation between gene expression level (TPM) and dN/dS. A black dot indicates a single gene. A blue line indicates a regression line. Rho and *P*-values of Spearman’s correlation test are also shown. **B** Comparison of the natural logarithm of dN/dS plus 1 among genes with H3K4me3 peaks within the promoter, exon, intron, and/or TTS regions (“Gene” in the figure), genes with peaks only in the intergenic regions (“Intergenic” in the figure), and genes without any surrounding histone modification peaks (“No peaks” in the figure). **C** Comparison of the natural logarithm of dN/dS plus 1 among genes with ATAC-seq peaks within the promoter, exon, intron, and/or TTS regions (“Gene” in the figure), genes with peaks only in the intergenic regions (“Intergenic” in the figure), and genes without any surrounding histone modification peaks (“No peaks” in the figure). Asterisks in (**B**) and (**C**) indicate *P* < 0.001 in the Wilcoxon rank sum test. H3K4me3 data of Gacu3 and ATAC-seq data of Gacu6 are shown here, whereas other data are shown in Fig. S4

Because gene expression levels are generally negatively associated with dN/dS values [68], we next tested the association between histone modifications and the dN/dS values of the nearest gene. We found a negative correlation between gene expression levels and dN/dS values in the stickleback (Fig. 4A). Next, we found that genes with H3K4me3 peaks at the gene exhibited significantly lower dN/dS values than genes without any peaks (Fig. 4B). Similarly, genes with ATAC-seq peaks exhibited lower dN/dS values than other genes (Fig. 4C). No apparent association was observed for H3K4me1, H3K9me3, and H3K27me3 (Fig. S4).

**Fig. 5:**
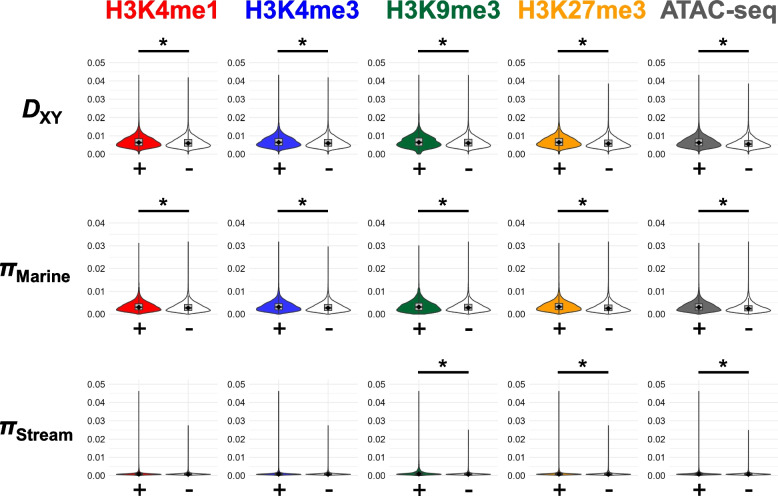
Comparison of *D*_XY_ and *π* between genomic regions within peaks ( +) and outside peaks ( −). Asterisks indicate *P* < 0.001 in the Wilcoxon rank sum test. Gill data of the Gacu3 individual is shown, whereas other data are shown in Fig. S5

Subsequently, we explored whether various histone modifications were associated with nucleotide variations within populations (*π*) and nucleotide diversity (*D*_XY_) between marine and stream populations. Both *π* and *D*_XY_ were higher in windows with peaks of any histone modifications or ATAC-seq than in windows without the peaks (Fig. 5, Fig. S5).

### Identifying possible *cis*-regulatory mutations

To examine whether the analysis of histone modifications helps identify candidate *cis*-regulatory mutations, we first conducted RNA-seq of gill tissues from marine and stream ecotypes. Principal component analysis (PCA) of all genes revealed three clusters (Fig. S6A), each corresponding to marine fish in seawater conditions, marine fish in freshwater conditions, and stream fish in freshwater conditions. PC1 explained 44.1% of the variance in the transcriptome, which separated the two ecotypes. PC2 explained 20.8% of the variance in the transcriptome, which reflected changes in gene expression of the marine ecotype between salinity conditions.

**Fig. 6:**
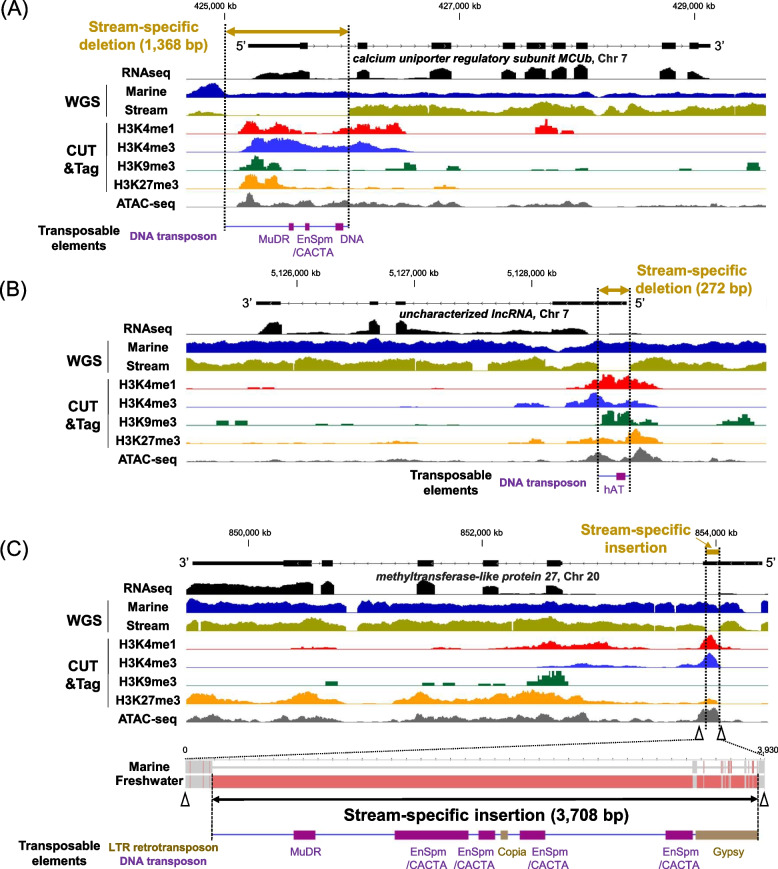
Ecotype-specific indels associated with transposable elements in the *cis*-regulatory regions with histone modification peaks. **A ***Calcium uniporter regulatory subunit MCUb* (LOC120821952). **B ***Uncharacterized lncRNA* (LOC120822152). **C ***Methyltransferase-like protein 27* (LOC120811024). RNA-seq (Marine-SW1), whole genome sequence (WGS; JR1_POf & Asutani 7), CUT&Tag (Gacu3), and ATAC-seq data (Gacu6) of the gills are visualized. Arrowheads in (**C**) indicate the locations of primers for genomic PCR. Above each panel, there is a gene model based on the version 5 stickleback reference genome. In the *Methyltransferase-like protein 27* gene (**C**), the stream ecotype contained an insertion sequence of 3,708 bp (LC797960)

Next, using these RNA-seq data, we screened for genes specifically expressed in the marine ecotypes. Forty-three genes met the criteria described in the Methods section (Fig. S6B). By checking the mapped short reads of whole genome sequencing of the marine ecotype, we found that 14 genes lacked the gene body, either the entire gene or the majority of the gene, in the marine ecotype. After excluding these 14 genes, 11 lacked the gene body in the stream ecotype, indicating that the marine-specific expression of these genes is simply due to the absence of the gene bodies in the stream ecotype rather than *cis*-regulatory differences. Among the remaining 18 genes, 13 exhibited clear H3K4me1 and/or H3K4me3 marks in the upstream region when we checked the short-read mapping of CUT&Tag data. Short-read mapping of the whole genome sequences of the stream ecotype revealed deletions of the possible *cis*-regulatory regions with H3K4me1 and H3K4me3 marks in three genes (Fig. 6): *calcium uniporter regulatory subunit MCUb* (Fig. 6A; 1,368 bp; NCBI Gene ID: 120,821,952), *uncharacterized lncRNA* (Fig. 6B; 272 bp; NCBI Gene ID: 120,822,152), and *methyltransferase-like protein 27* (Fig. 6C; 117 bp; NCBI Gene ID: 120,811,024).

To validate stream ecotype-specific deletions, we conducted genomic PCR. We confirmed stream-specific deletions in *calcium uniporter regulatory subunit MCUb* (Fig. 6A) and *uncharacterized lncRNA* (Fig. 6B)*,* as expected (Fig.S6C). In the *MCUb* gene, the region deleted in the stream ecotype contained sequences similar to the MuDR DNA transposon, En/Spm DNA transposon, and another DNA transposon (DNA-2-15_DR) in the marine ecotype (Fig. 6A; Table S5). In the *lncRNA* gene, the region deleted in the stream ecotype contained sequences similar to the hAT DNA transposon in the marine ecotype (Fig. 6B; Table S5). In contrast, we found that a 3,708 bp insertion (accession number: LC797960) replaced a 5’-non-coding region of the *methyltransferase-like protein 27* gene in the stream ecotype. This stream ecotype-specific insertion replaced the region where the H3K4me1 and H3K4me3 marks were present in the marine ecotype (Fig. 6C). This stream-specific insertion also contained sequences homologous to transposable elements, including the MuDR DNA transposon, En/Spm DNA transposon, Copia retrotransposon, and Gypsy retrotransposon (Fig. 6C; Table S5).

## Discussion

Here, we showed that the analysis of histone modifications can contribute to the identification of candidate *cis*-regulatory elements. Genes with active marks, such as H3K4me1, H3K4me3, and open chromatin were highly expressed. In contrast, the expression of genes containing the repressive mark, H3K27me3 was reduced. These data suggest that our chromatin analysis worked on the stickleback, except for the samples using H3K9me3 antibody or samples from frozen tissues. Consistent with the gene expression patterns, the protein sequences of genes with nearby H3K4me3 peaks were relatively conserved compared with those without peaks. Notably, we found that genomic regions with any peaks of histone modifications show higher *π* and *D*_XY_ than genomic regions without the peaks. These results indicated that these regions have high mutation rates. Although we do not know what causes this pattern, chromatin states generally influence mutation rates [69]. While the genomic regions with histone modifications displayed higher mutation rates, the genes with active H3K4me3 marks had lower protein sequence evolution (lower dN/dS), suggesting selection preserving amino acid sequence despite possibly higher dS. Furthermore, recent genome-wide association studies of complex phenotypic traits in humans have shown that significant variants are enriched in regions of active chromatin such as promoters and enhancers [70]. Therefore, variants within peaks of histone modifications may also contribute to evolution in nature. Further studies on the association among histone modifications, mutation rates, and phenotypic variations will help understand the causes of variations in mutation rates across the genome.

We also identified indels in the potential *cis*-regulatory regions. Because the inserted sequences contained transposons, these indels may have been caused by transposon activity. Several studies have shown that transposon insertions can alter gene expression patterns, such as those involved in coloration and pigmentation in animals [71], including fish [72] and butterflies/moths [73–75]. Our results indicate that the analysis of histone modifications can contribute to the identification of *cis*-regulatory mutations. Here, we identified three potential indels that may influence differential gene expression between ecotypes. The *MCUb* gene product regulates calcium uptake in the mammalian mitochondria [76]. Because mitochondria play important roles in fish osmoregulation [77, 78] and the expression levels of genes involved in mitochondrial functions are altered by salinity in the stickleback [79], the ecotypic difference in *MCUb* expression may be adaptive. The other two genes are involved in epigenetic regulation [80, 81], although precise physiological functions are unknown. Because epigenetic regulation is also important for osmoregulation [82], further studies on the functions of these genes will contribute to a better understanding of the genetic mechanisms of environmental adaptation.

In our analysis, transcription start sites (TSS) were not always marked with active promoters. Because we used bulk tissues composed of multiple cell types, cell type-specific epigenetic signals may have been masked by those of other cell types. Single cell ATAC-seq and CUT&Tag technologies have recently been developed [83], which will be useful in resolving this potential issue. Additionally, when the annotation of the TSS in the genome assembly is incorrect, CUT&Tag for identifying active promoters will improve gene annotation.

The ENCODE project has mapped functional sites within the non-coding regions of the human genome [84, 85]. Similarly, we can map the functional elements in the non-coding regions of natural organisms. Recently, genome editing using CRISPR-Cas9 techniques has been developed and improved [86]. Applying these techniques to the candidate *cis*-regulatory regions will enable us to conduct functional validation of the identified mutations in the future. During field trips, it is often difficult to perform CUT&Tag experiments immediately after collecting fresh samples. Here, we tested whether CUT&Tag using frozen tissues gave rise to similar results to those with fresh samples. Unfortunately, as frozen tissues made a cluster different from fresh samples (Fig. S2), further improvement and refinement are needed to use frozen samples for CUT&Tag.

## Conclusions

We have demonstrated that mapping and analyzing histone modifications can help identify *cis*-regulatory elements in the threespine stickleback and accelerate the identification of causative mutations in the non-coding regions underlying naturally occurring phenotypic variations. Further mapping and analysis of chromatin states in natural populations will improve our understanding of the evolution of *cis*-regulatory elements that can cause divergent adaptation in nature.

### Supplementary Information

Supplementary Material 1.

Supplementary Material 2.

## Data Availability

Illumina reads have been deposited at the DDBJ/NCBI under BioProject PRJDB17468 (https://www.ncbi.nlm.nih.gov/bioproject/PRJDB17468). The identified stream-specific insertion sequence is available from https://www.ncbi.nlm.nih.gov/nuccore/LC797960.
