# Supplementary material for: Genome-wide analysis of histone modifications can contribute to the identification of candidate cis-regulatory regions in the threespine stickleback fish
Source: BMC Genomics. 2024 Jul 11;25:685. doi: 10.1186/s12864-024-10602-w (PMC11241946; doi:10.1186/s12864-024-10602-w)
Supplement: Supplementary file 2 — Supplementary Material 2. [file 12864_2024_10602_MOESM2_ESM.pdf]

Figure S1

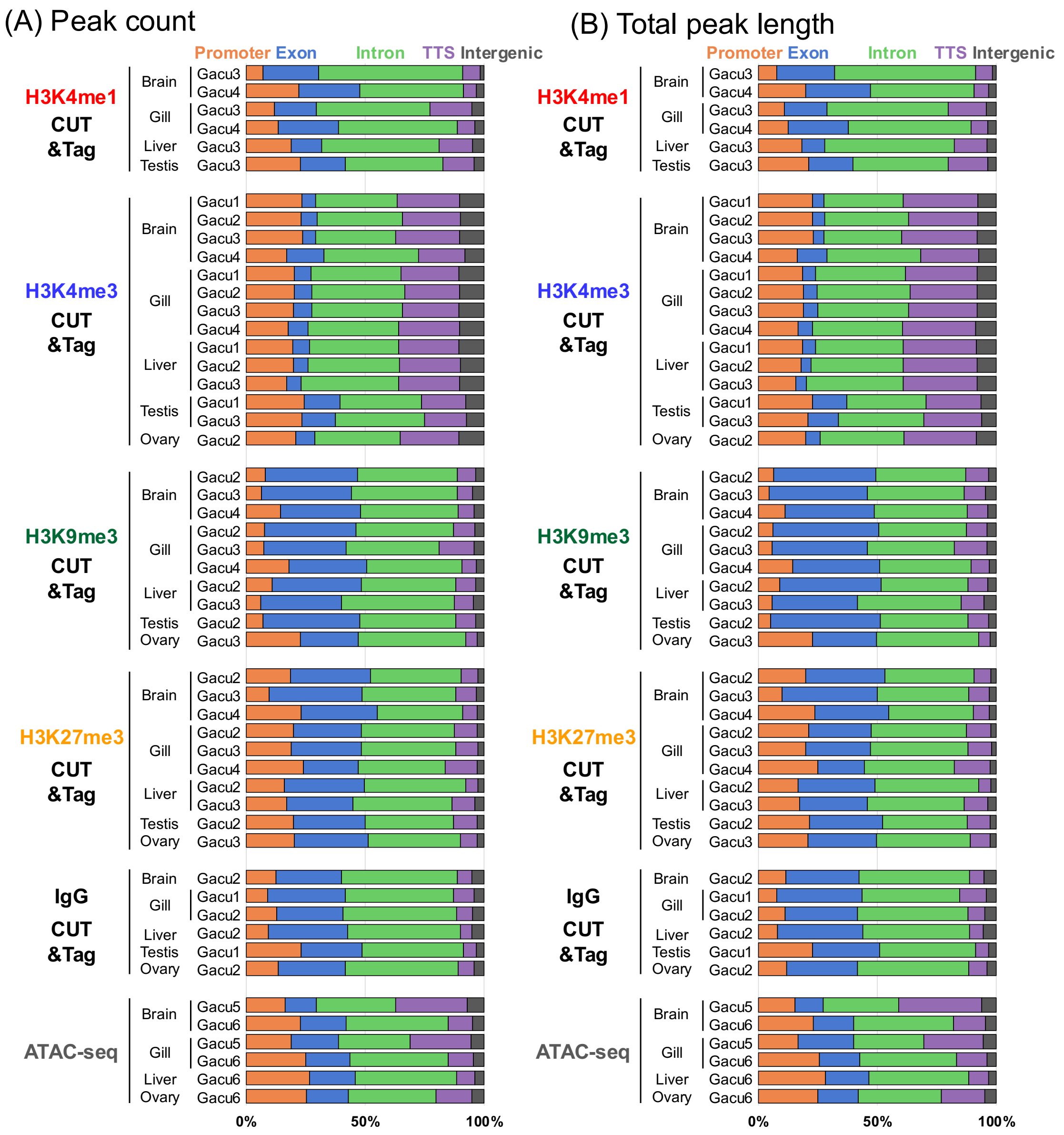

Figure S1. Locations of the peaks of CUT&Tag and ATAC-seq in the genome. (A) Proportions of peak counts in different genomic regions: promoter, exon, intron, TTS, and intergenic regions. (B) Proportions of peak length in different genomic regions: promoter, exon, intron, TTS, and intergenic regions.

Figure S2

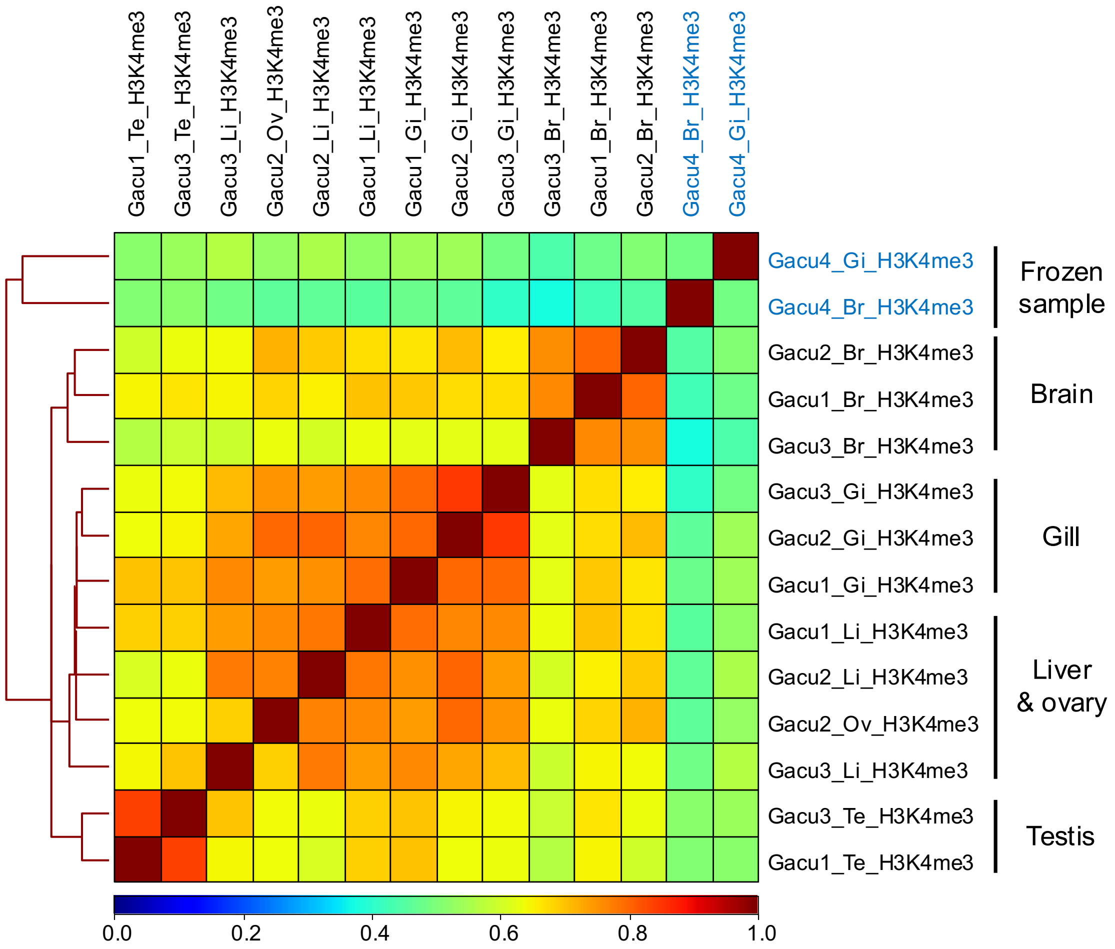

Figure S2. Clustering analysis of CUT&Tag data. Heatmap and dendrogram using Spearman's correlation coefficients are shown. Colored squares indicate the Spearman's correlation coefficients (see the scales below). Sample names with blue letters are frozen samples.

Figure S3

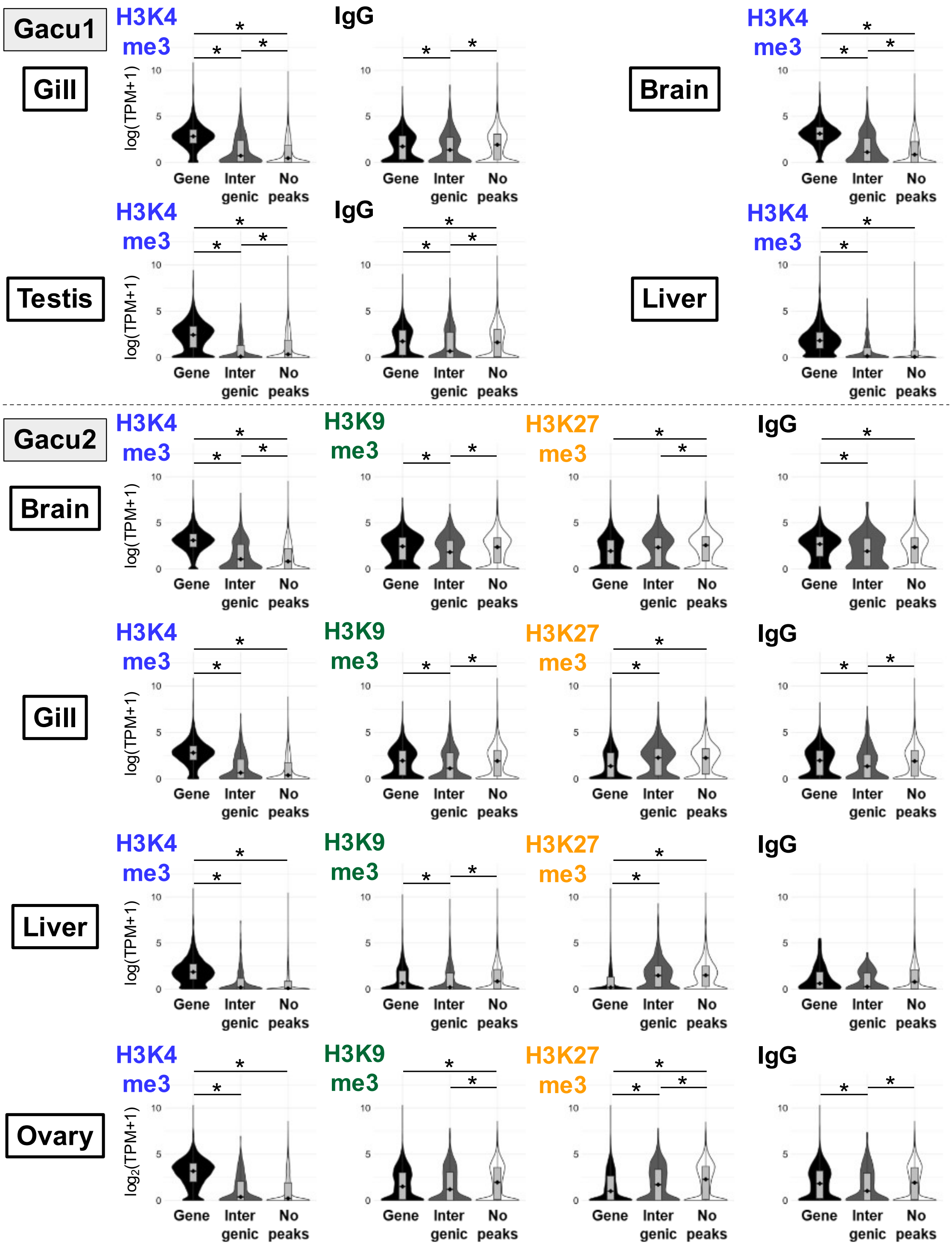
Gacu1
Gill
Testis
Brain
Liver
Gacu2
Brain
Gill
Liver
Ovary
H3K4 me3
IgG
H3K9 me3
H3K27 me3
log(TPM+1)
log2(TPM+1)
Gene
Inter genic
No peaks
*

Figure S3 (Cont.)

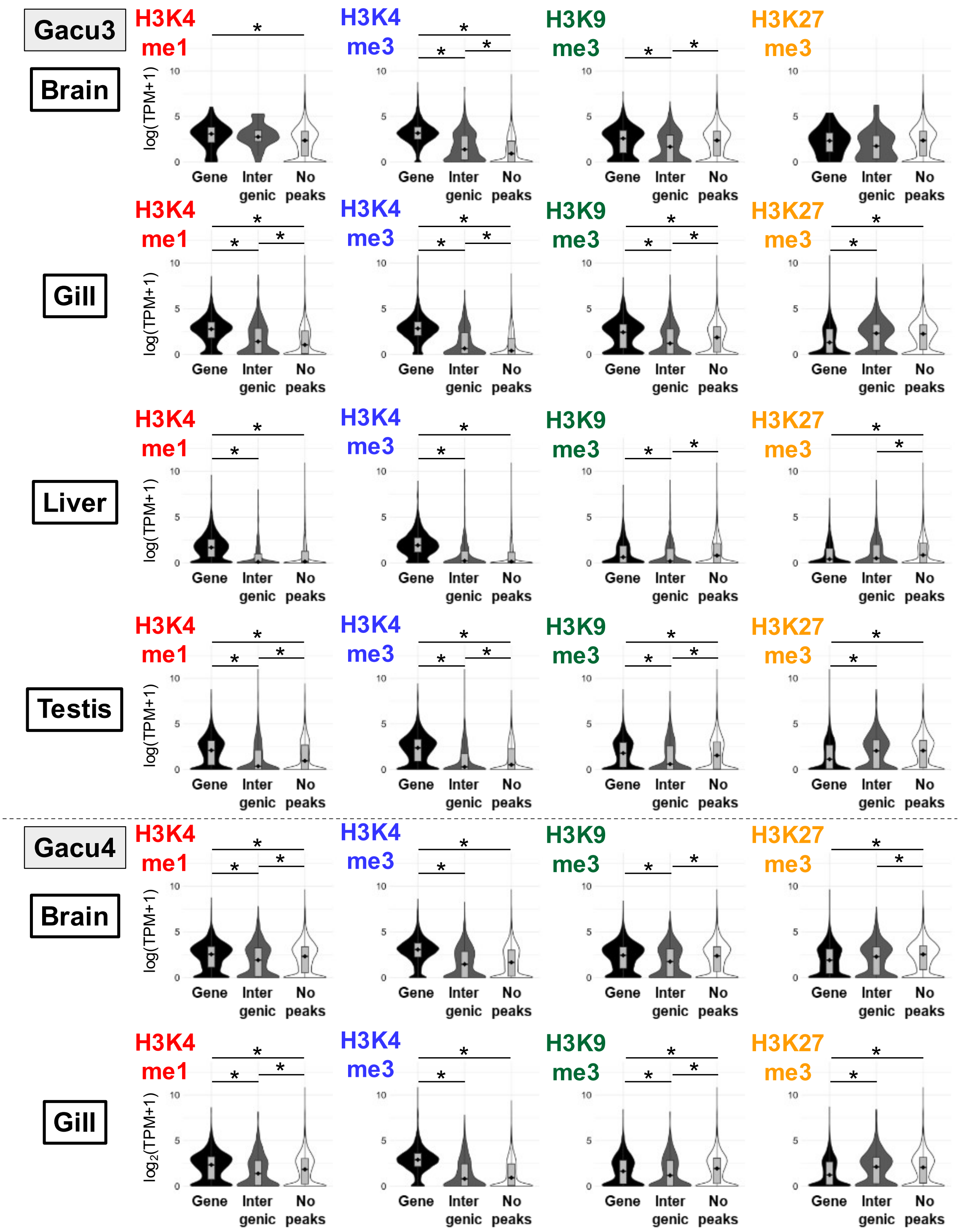

Figure S3 (Cont.)

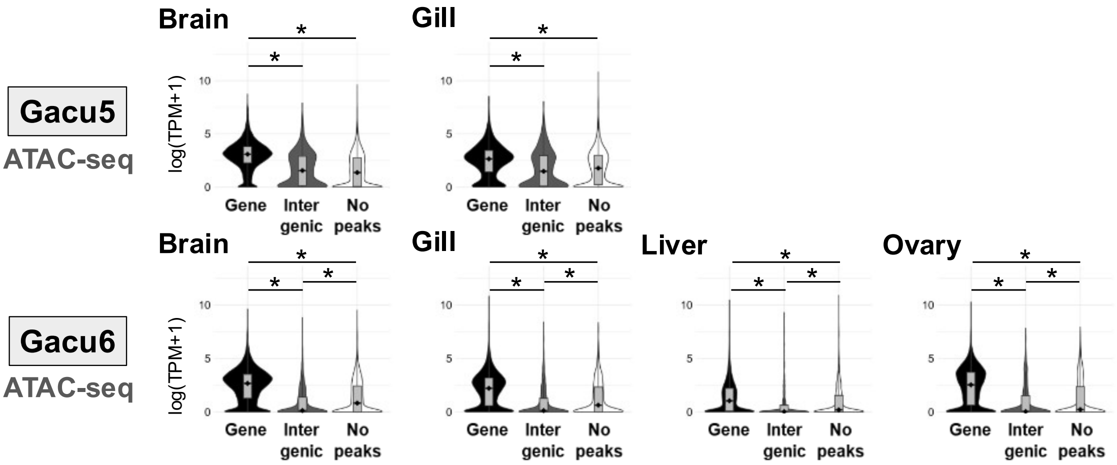

Figure S3. Association between histone modifications and gene expression levels. All genes were classified into the following three categories: genes with peaks within the promoter, exon, intron, and/or TTS regions (“Gene” in the figure), genes with peaks only in the intergenic regions (“Intergenic” in the figure), and genes without any surrounding histone modification peaks (“No peaks” in the figure). Asterisks indicate $P < 0.001$ in the Wilcoxon rank sum test. The Y-axis indicates the natural logarithm of TPM plus 1.

Figure S4.

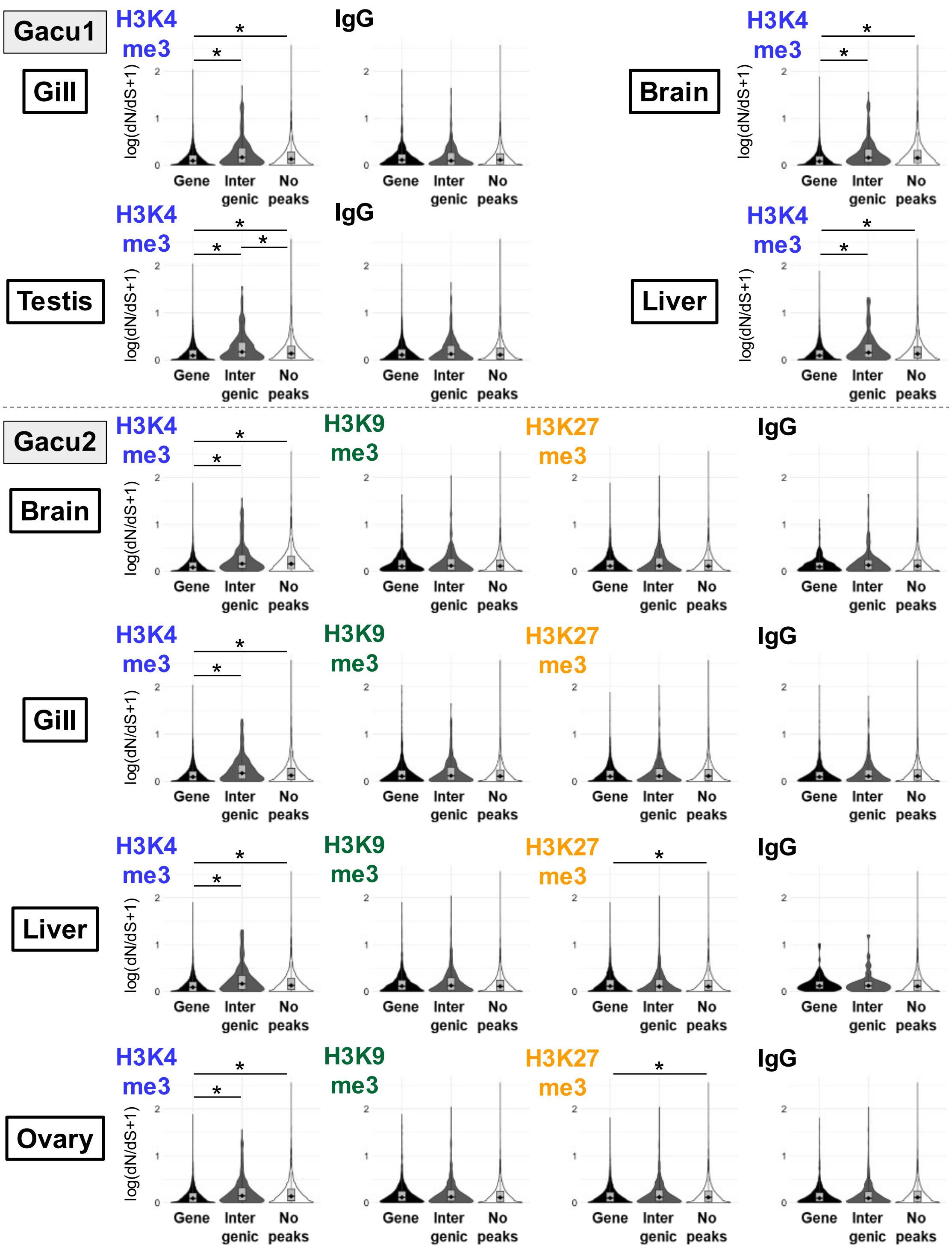
Gacu1
Gill
Brain
Testis
Liver
Gacu2
Brain
Gill
Liver
Ovary
H3K4 me3
H3K9 me3
H3K27 me3
IgG
log(dN/dS+1)
Gene
Inter genic
No peaks
*

Figure S4 (Cont.)

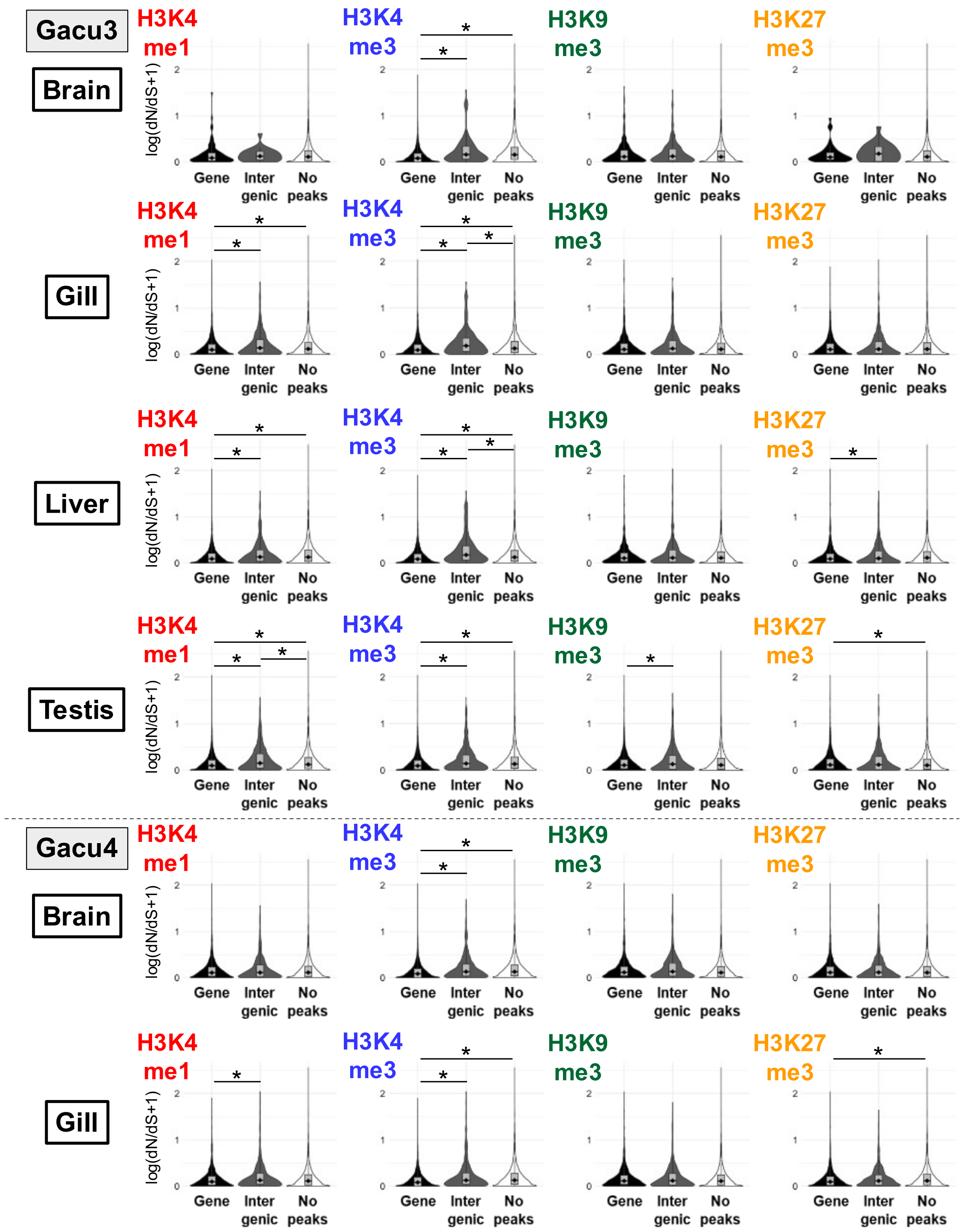

Figure S4 (Cont.)

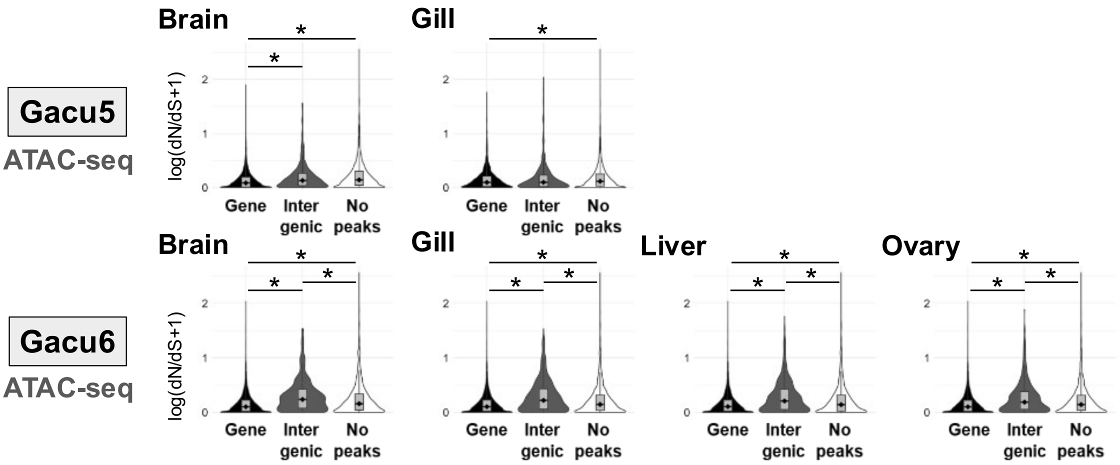

Figure S3. Comparison of the natural logarithm of dN/dS plus 1 among genes with peaks within the promoter, exon, intron, and/or TTS regions (“Gene” in the figure), genes with peaks only in the intergenic regions (“Intergenic” in the figure), and genes without any surrounding histone modification peaks (“No peaks” in the figure). Asterisks indicate $P < 0.001$ in the Wilcoxon rank sum test.

Figure S5.

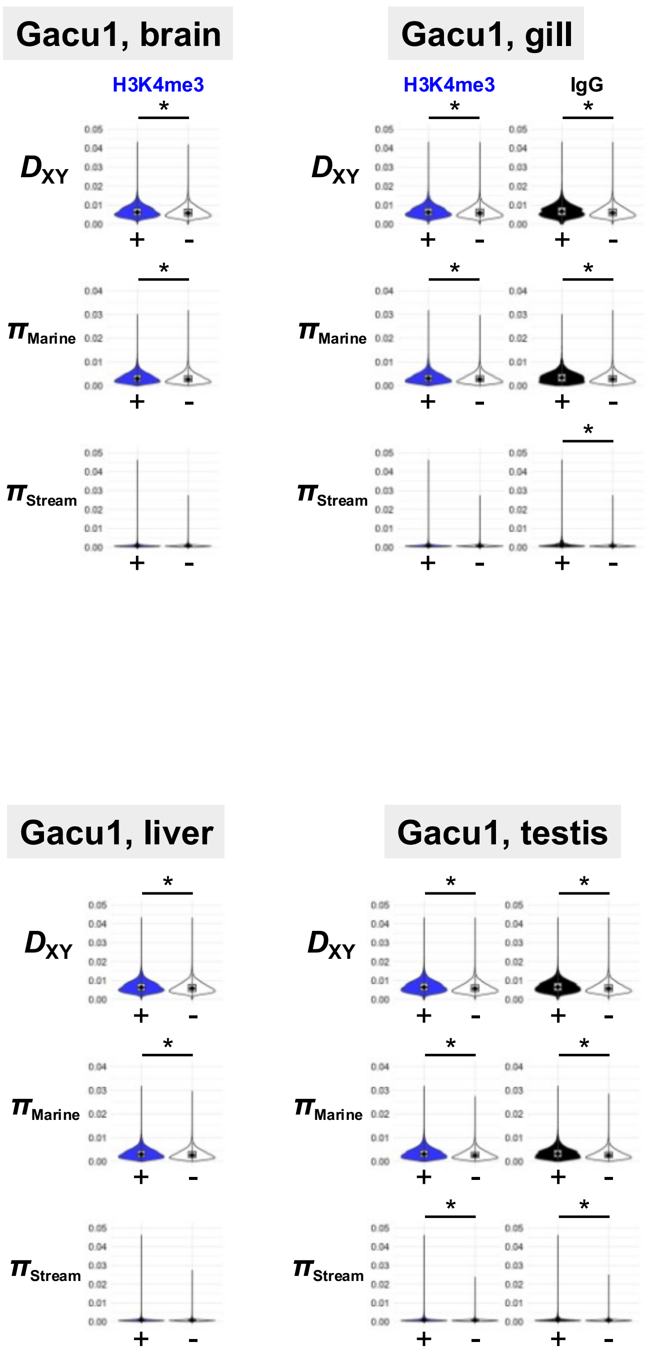
Gacu1, brain
H3K4me3
D_{XY}
π_{Marine}
π_{Stream}
Gacu1, gill
H3K4me3
IgG
Gacu1, liver
Gacu1, testis

Figure S5 (Cont.)

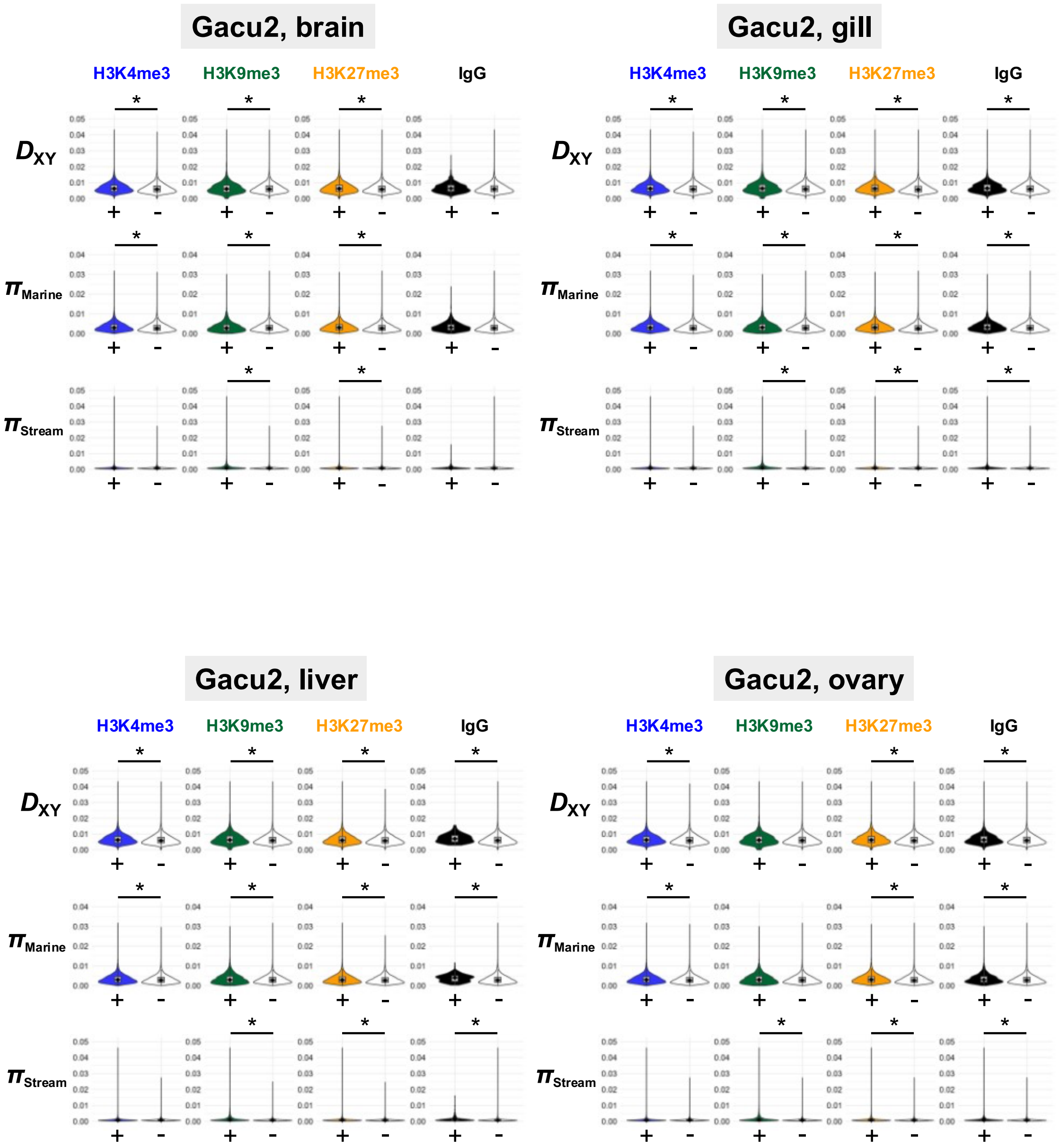

Figure S5 (Cont.)

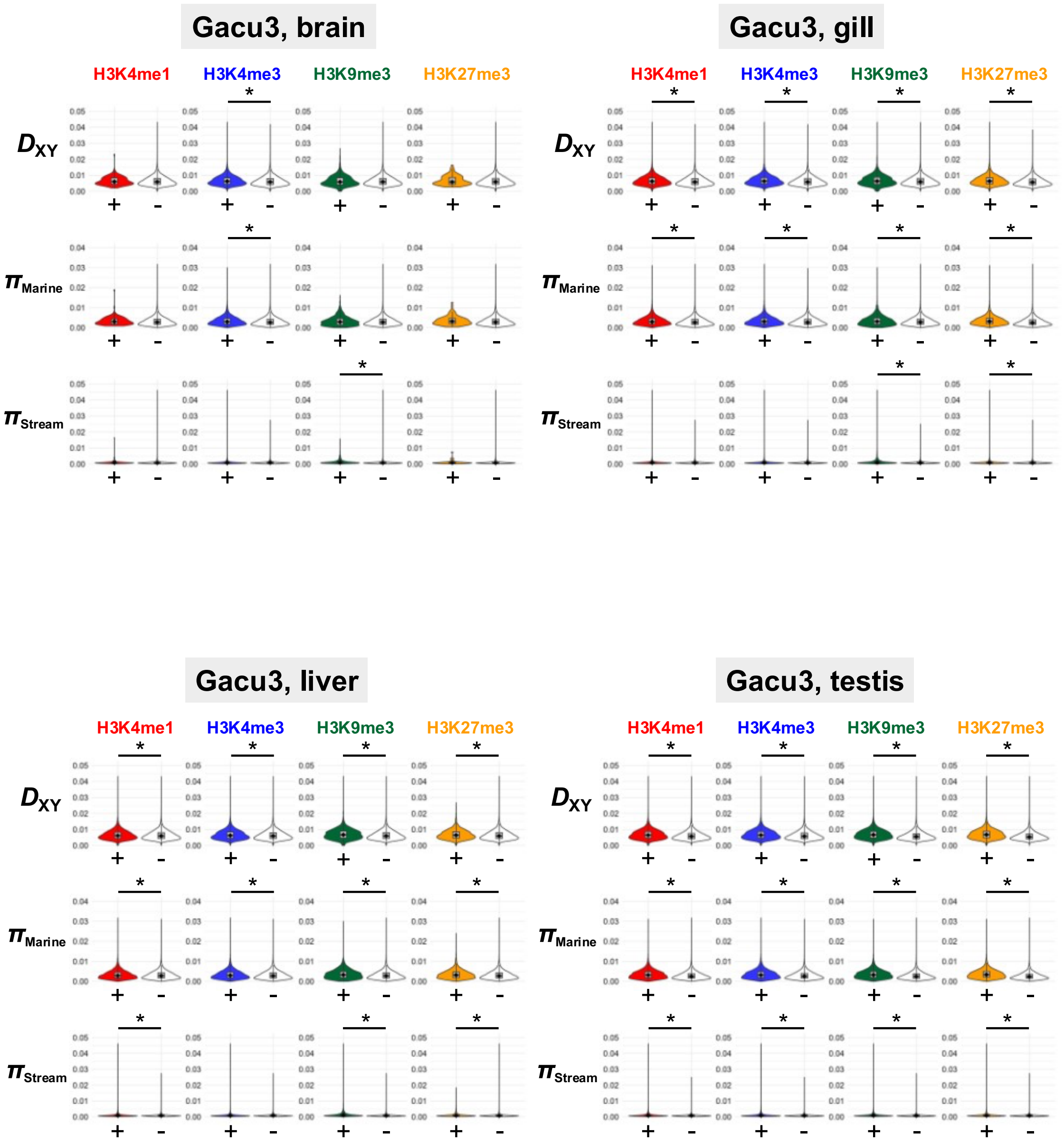

Figure S5 (Cont.)

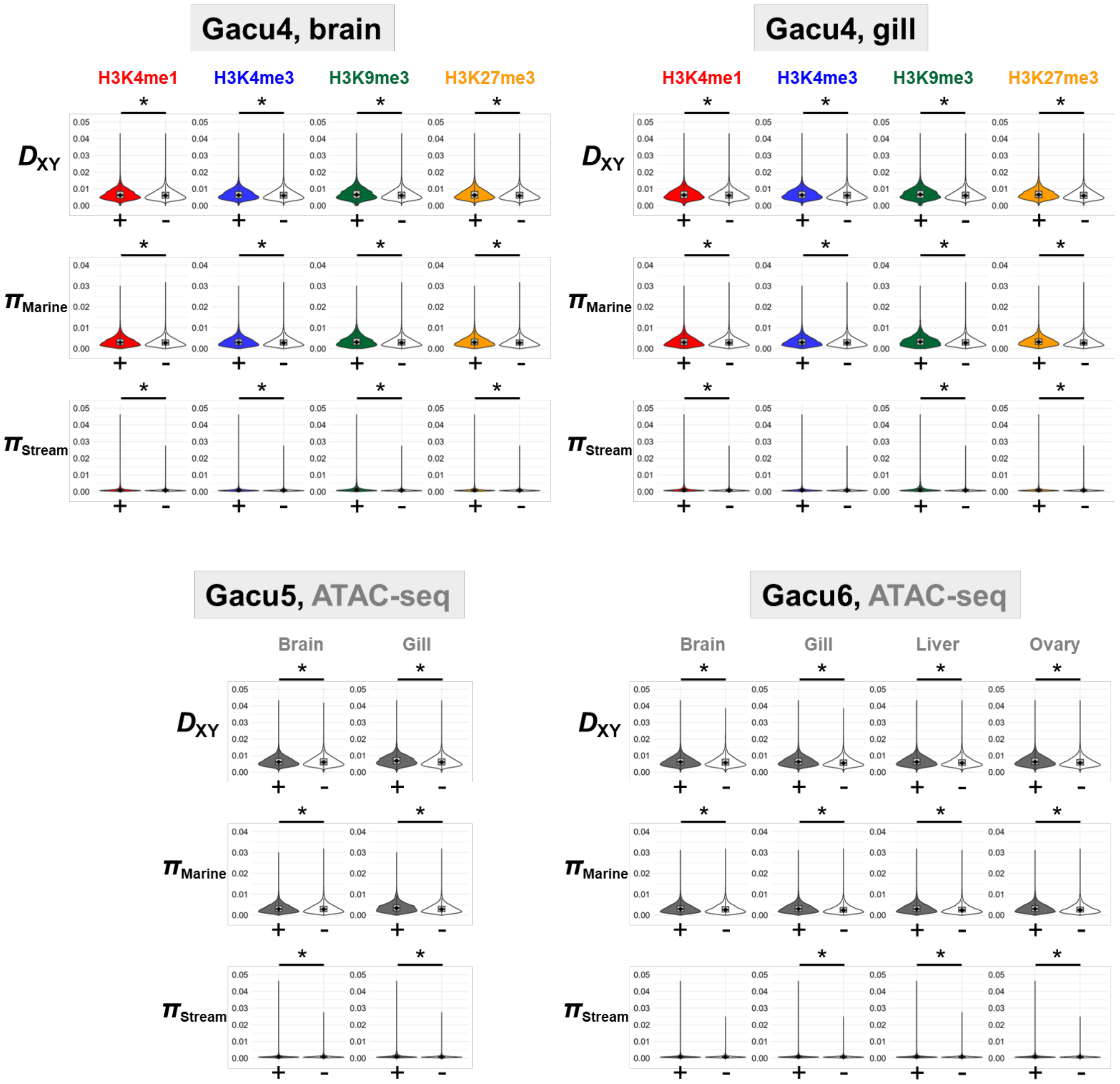

Figure S5. Comparison of D_{XY} and π between genomic regions within peaks (+) and outside peaks (-). Asterisks indicate $P < 0.001$ in the Wilcoxon rank sum test.

Figure S6.

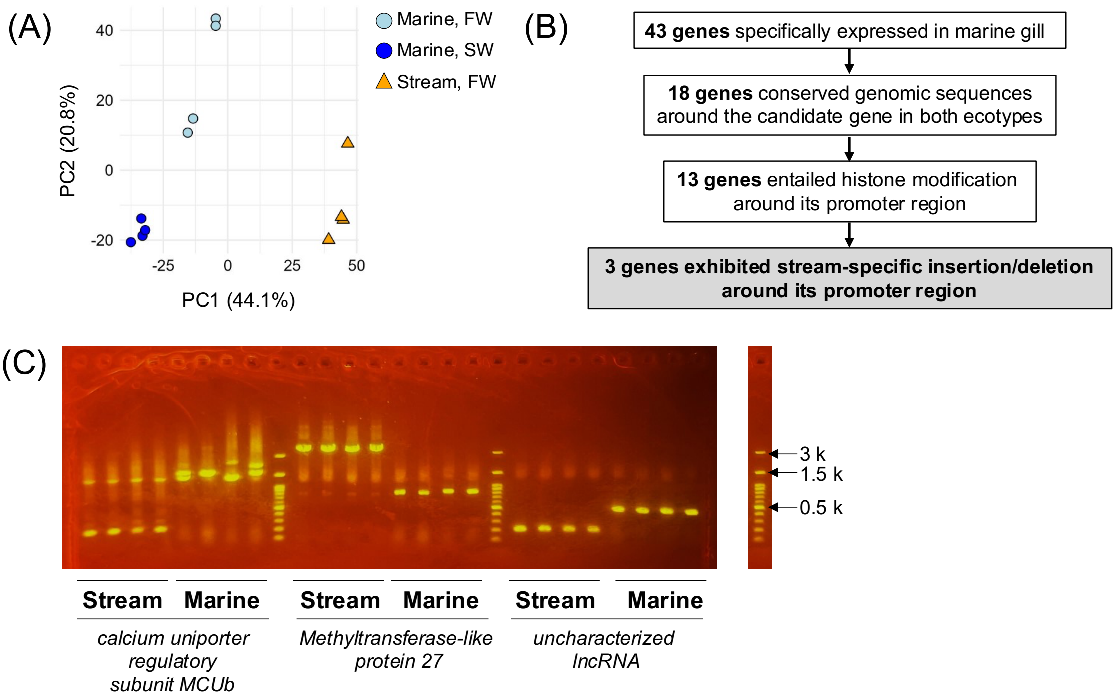

Figure S6. Transcriptomic analysis and genomic-PCR for identifying ecotype-specific indels. (A) Principal component analysis (PCA) of marine and stream ecotypes reared in freshwater or seawater conditions. All genes were used for PCA. (B) Outline of the procedures for identifying candidate genes for which gene expression changes are caused by ecotype-specific indels. (C) Electrophoresis images of genomic PCR products with primers flanking the candidate indel regions identified using short-read mapping. PCR products were run on 1% agarose gel and visualized with Midori Green Xtra (FastGene, Tokyo, Japan). Four individuals of the marine ecotype and four individuals of the stream ecotype were analyzed.
